# Comparison of Ultrasonic and Pneumatic Intracorporeal Lithotripsy Techniques during Percutaneous Nephrolithotomy

**DOI:** 10.1155/2013/604361

**Published:** 2013-08-18

**Authors:** Tolga Karakan, Akif Diri, Ahmet Metin Hascicek, Berat Cem Ozgur, Serkan Ozcan, Muzaffer Eroglu

**Affiliations:** Sağlık Bakanlığı Ankara Eğitim ve Araştırma Hastanesi Şükriye Mh., Ulucanlar Cd. No. 89, 06340 Ankara, Turkey

## Abstract

*Objectives*. To compare the effectiveness and safety of ultrasonic and pneumatic lithotripters in the treatment of renal stone disease. *Materials and Methods*. A total of 227 consecutive percutaneous nephrolithotomy procedures for renal calculi were performed. In 107 patients ultrasonic lithotriptors were used (group I) and in 83 patients pneumatic lithotriptors were used (group II). In the remaining 37 patients, stones were managed with both pneumatic and ultrasonic lithotripters. Follow-up studies included intravenous urography (IVU) and/or computed tomography (CT). *Results*. The mean operative time and duration of hospitalization were similar between the groups. In the ultrasonic treatment group, 100 (96.9%) patients were stone-free on postoperative day 1 and 5 (4.6%) went on to undergo an additional treatment modality, resulting in a total stone-free rate of 97.2%. In the pneumatic lithotripsy group, 68 (81.9%) patients were stone-free after the primary procedure on the first day and 15 (18.1%) went on to undergo an additional treatment modality, resulting in a stone-free rate of 91.5%. The final stone-free rates at 3 months postoperatively in groups I, II, and III were 97.2%, 91.5%, and 87.9%, respectively (P = 0.826). *Conclusions*. We conclude that both ultrasonic and pneumatic lithotripters are effective and safe for intracorporeal lithotripsy. However, the ultrasonic lithotripter provides higher stone-free rates with similar morbidity compared with pneumatic devices.

## 1. Introduction

Percutaneous nephrolithotomy (PNL) has become the preferred method for treatment of large renal calculi since this modality was first utilized in 1976 by Fernström and Johanson [1]. This technique has the advantages of higher stone-free rates, cost effectiveness, and early convalescence compared with other modalities such as shock wave lithotripsy (SWL) and open surgery [2, 3]. Intracorporeal lithotripsy is one of the most important steps that affect the success rate of this surgical method, and for this step pneumatic and ultrasonic lithotripters are commonly used energy sources [3]. In this study, we aimed to compare the success rates of pneumatic and ultrasonic lithotripsy techniques during PNL.

## 2. Patients and Methods

### 2.1. Patients

A total of 227 consecutive PNL procedures for renal calculi were performed at our institution between 2009 and 2012. In 107 patients (47.1%, mean stone size 34 mm^2^) ultrasonic lithotriptors were used (group I) and in 83 patients (36.5%, mean stone size 50 mm^2^) pneumatic lithotriptors were used (group II). In the remaining 37 patients (16.3%, mean stone size 72 mm^2^), stones were managed with both pneumatic and ultrasonic lithotripters (group III). 

Patients under 18 years of age and patients with multicalyceal stones requiring multitract PNL were excluded from the study. Patient- and procedure-related factors and perioperative and postoperative variables, such as the operation and fluoroscopy times, success rates, and hospitalization time, were compared between the groups. 

The patient assessment included medical history, physical examination, complete blood count, serum biochemistry, coagulation tests, urinalysis, urine culture, intravenous urography (IVU), and/or computed tomography (CT). The stone location was identified using preoperative CT or IVU, and the size was calculated according to the European Association of Urology guidelines [4]. Positive urine cultures were adequately treated with appropriate antibiotics, and all patients had a negative culture before surgery.

### 2.2. Operative Technique

A standardized PNL procedure was performed in all cases as described previously [5]. Briefly, a 5F or 6F ureteral catheter was initially placed in a lithotomy position under general anesthesia. After contrast injection through the ureteral catheter, an 18-gauge needle was passed under the fluoroscopic guidance. Following the urine coming out through the needle, a 0.038 inch guidewire was passed through the needle to the collecting system. Dilation was performed using balloon dilators to 30 Fr. Fragmentation and stone removal were accomplished in all patients using pneumatic or ultrasound energy and retrieval graspers through a rigid 26 Fr nephroscope. EMS Swiss Lithoclast was used for pneumatic lithotripsy, at a pressure of 3 atm and a frequency of 12 Hz. For the ultrasonic lithotripsy, EMS lithotripter was used at the maximum levels of the (100%) settings. The ultrasonic probe was passed through the nephroscope, and the stone was trapped between the probe and the urothelium. To maximize lithotripsy efficiency, the physician moved the ultrasonic probe over the stone surface in a “painting” fashion. At the conclusion of the procedure, an 18 Fr nephrostomy tube was placed in all cases, which was removed on postoperative days 1-2, and the patient was discharged to home the next day.

### 2.3. Data Analysis

Plain film was performed after one day to evaluate stone-free rate. Treatment success was defined as stone-free or clinically insignificant residual fragments (residual fragments ≤3 mm). Residual stones were managed by second-look PNL or SWL. Stone-free status was reevaluated after 3 months with IVU or CT in an outpatient clinic setting. Statistical analysis was performed using the one-way ANOVA *t*-test and chi-square test. All results were reported as means ± standard deviation, and *P* < 0.05 was considered statistically significant.

## 3. Results

### 3.1. Patient and Stone Characteristics

A retrospective review was identified in 227 patients, including 139 males (61%) and 88 females (39%). The mean patient age was 48 ± 15 years (18–81 years). The stone was on the right side in 109 patients (48%) and on the left side in 118 patients (52%). The mean stone size was 46 ± 33 mm^2^ (11–262 mm^2^). The patient age, male/female ratio, and stone location were similar in each group (*P* = 0.463, *P* = 0.698, and *P* = 0.334, resp.). However, the mean stone size was 33.8 mm^2^ (11.5–78.8) in group I, 49.9 mm^2^ (16.9–262.5) in group II, and 72.3 mm^2^ (28.8–151.8) in group III. As delineated in Table 1, mean stone size was significantly larger in patients who were treated with a combination of ultrasound and pneumatic lithotripters (*P* = 0.001). 

### 3.2. Operative Findings and Postoperative Data

The mean operative time, fluoroscopic screening time, and duration of hospitalization were similar between the groups (*P* > 0.05 for each parameter). In the ultrasonic treatment group, 100 (96.9%) patients were stone-free on postoperative day 1and 5 (4.6%) went on to undergo an additional treatment modality, resulting in a total stone-free rate of 97.2%. In the pneumatic lithotripsy group, 68 (81.9%) patients were stone-free after the primary procedure on postoperative day 1 and 15 (18.1%) went on to undergo an additional treatment modality, resulting in a stone-free rate of 91.5%. The final stone-free rates at 3 months postoperatively in groups I, II, and III were 97.2%, 91.5%, and 87.9%, respectively (*P* = 0.826). Additional treatment modalities, including SWL and second-look PNL, were performed on 5 patients in group I, 15 patients in group II, and 4 patients in group III (*P* = 0.008).

Most of the complications were minor, and no difference was observed between the groups in either intra- or postoperative complications. The main complications were fever, pain, mild bleeding, urinary leakage, and postoperative infections. All these patients were treated conservatively with analgesics, antibiotics, and/or prolonged double-j stent placement. No patient in either of the groups required a blood transfusion.

## 4. Discussion

Renal stone treatment options have changed dramatically during the last two decades with the technological advancement of instruments [6]. Today PNL is the first choice treatment modality for most renal stones larger than 2 cm, multiple renal stones, and also for complex renal calculi [7]. The introduction and advances in various forms of intracorporeal lithotripters, especially ultrasonic and pneumatic devices, have improved the stone-free rates after PNL, while concomitantly decreasing the risk of complications. 

These various intracorporeal lithotripters work on different physical principles of stone fragmentation [8]. Pneumatic lithotripters work on the same principle as collision with a bullet; on impact, energy transmits compressed air pulses within a steel probe to the calculi to be fragmented [9]. This technique offers safe, cheap, and effective clearance of calculi, and it is particularly useful for large and hard stones. Also, all stones can be destroyed regardless of their composition, but subsequent extraction of the stone fragments is required [10, 11]. According to the literature, the success rate of pneumatic lithotripsy appears to be higher than 84% [5, 10–12]. In this study, we achieved an overall success rate of 90.8%, which is similar to that in the literature regarding the general results of PNL. Our results show the effectiveness and safety of this technique.

Ultrasonic lithotripsy is still the most commonly used lithotripter with rigid nephroscopes during PNL [13]. It fragments stones into small pieces and has the ability to aspirate these particles through the hollow bore of the transducer, which eliminates manual stone extraction [10, 11]. This technique was the standard method of lithotripsy for many years, with a fragmentation rate of 97% [10]. Although this lithotripsy technique has high success rates, it is not universally successful, especially in the setting of hard stones, such as calcium oxalate monohydrate and cysteine [14]. Another disadvantage is the potential for overheating due to conversion of vibration energy to heat energy [14]. Nevertheless, overheating of the probe can cause tissue injury. In a rat model, Diri et al. noted that ultrasonic devices have a potential risk for tissue injury [11]. They showed a significant increase in inflammation, papillary projection, stratification, and microscopic or macroscopic stone formation in the bladder wall of rats which was treated with ultrasonic lithotripsy. 

In the present study, we compared the efficacy and safety of the standard ultrasonic device with those of a pneumatic lithotripter and the combined use of pneumatic and ultrasonic devices. There were no significant differences in the complication rates, mean operative times, and mean hospitalization times between the three groups. However, there was a higher percentage of stone-free patients in the ultrasonic lithotripsy group than pneumatic and combined lithotripsy groups.

This series has some limitations, including its retrospective nature and relatively small number of patients. However, the most important limitation of the present study is that there was a significant difference in stone size between the 3 groups. Therefore, our findings must be confirmed by further prospective randomized studies.

## 5. Conclusion

In the present study, pneumatic and ultrasonic lithotripters were compared, and both of them were found to be effective, safe, and reliable management modalities. However, the ultrasonic lithotripter provided higher stone-free rates with similar morbidity compared with pneumatic devices. 

## Figures and Tables

**Table 1:** Demographic data, stone, and operative characteristics.

	Group I	Group II	Group III	*P* value
Number of patients (%)	107 (47.1%)	83 (36.5%)	37 (16.3%)	
Median age, years	50	47	48	0.463
Male/female	62/45	54/29	23/14	0.698
Mean stone size (range) mm^2^	33.8 (11.5–78.8)	49.9 (16.9–262.5)	72.3 (28.8–151.8)	0.001*
Stone laterality				0.334
Right	50	46	22	
Left	57	37	15	
Stone-free rate (%)				
1 day postoperatively	96.9%	81.9%	78.3%	0.023*
3 months postoperatively	97.2%	91.5%	87.9%	0.826
Auxiliary procedure (%)	4.6%	18.1%	10.8%	0.008*

*Significant at 0.05 level.
